# Family history of breast cancer and young age at diagnosis of breast cancer increase risk of second primary malignancies in women: a population-based cohort study

**DOI:** 10.1038/sj.bjc.6603404

**Published:** 2006-10-03

**Authors:** M Prochazka, P Hall, F Granath, K Czene

**Affiliations:** 1 Department of Medical Epidemiology and Biostatistics, Karolinska Institutet, PO Box 281, SE-171 77 Stockholm, Sweden; 2 Department of Medicine at Karolinska University Hospital, Clinical Epidemiology Unit, SE-171 76 Stockholm, Sweden

**Keywords:** breast cancer, second primary cancer, familial risk

## Abstract

Among 152 600 breast cancer patients diagnosed during 1958–2000, there was a 22% increased risk of developing a second primary non-breast malignancy (standardised incidence ratio (SIR)=1.22; 95% confidence interval (CI): 1.19–1.24). The highest risk was seen for connective tissue cancer (SIR=1.78; 95% CI: 1.49–2.10). Increased risks were noted among women diagnosed with breast cancer before age 50. Oesophagus cancer and non-Hodgkin's lymphoma showed six- and four-fold higher risks, respectively, in women with a family history of breast cancer compared to those without in the ⩾10-year follow-up period.

With advances in early diagnosis and treatment, for many women breast cancer is becoming increasingly curable. Breast cancer survivors have been reported as having a 10–60% increased risk of a second primary malignancy at other sites compared to the general population (Mellemkjaer *et al*, 2006; Raymond and Hogue, 2006; Yu *et al*, 2006). Multiple primary tumours may be caused by shared aetiological factors (e.g. diet, reproductive history, hormonal status, and environmental exposure), inherited susceptibility, adverse effects of treatment, increased medical surveillance, and possible interactions between these factors (Travis, 2002; Suzuki *et al*, 2005). Up to 7% of breast cancer cases are estimated to be due to breast cancer susceptibility genes (e.g. *BRCA1*, *BRCA2*, *p53*, and *PTEN*) (Edlich *et al*, 2005), adding to the risk of other cancers (Edlich *et al*, 2005; McClain *et al*, 2005).

Most breast cancer patients will die of causes other than breast cancer; so the long-term effects of treatment have become correspondingly important (Shapiro and Recht, 1994). Increased risks of soft tissue sarcomas (Karlsson *et al*, 1998; Levi *et al*, 2003), lung (Prochazka *et al*, 2002, 2005), and oesophageal cancer (Zablotska *et al*, 2005) have been associated with radiotherapy of the breast. In addition, a causal relationship between tamoxifen and endometrial cancer risk has been established (Bergman *et al*, 2000; Swerdlow and Jones, 2005).

We aimed to examine the risk of second malignancies in women with breast cancer, according to age at and time interval since breast cancer diagnosis, and calendar period. In addition, we investigated the effect of family history of breast cancer.

## MATERIALS AND METHODS

Eligible patients included 171 386 women with histologically confirmed invasive breast cancer as the first cancer reported to the Swedish Cancer Registry between 1958 and 2000. The Swedish Cancer Registry receives reports on newly diagnosed cancers from both pathologists/cytologists and physicians. Reporting to the register is compulsory, and 96% of all cancers in Sweden are reported (Mattsson and Wallgren, 1984). Almost all diagnosed breast cancer patients in Sweden are treated within the national health care system and treatment regimens are similar throughout the country.

The breast cancer cohort was matched with the Swedish Emigration and Cause of Death Registries. Excluded were women diagnosed with a second cancer (*n*=7370) or dying (*n*=3547) within 1 month after the first primary cancer. Additional exclusions included women identified as immigrants as data on previous cancers were unavailable, and women who had emigrated before the diagnosis of breast cancer and left Sweden for ⩾1year (*n*=7883). Thus, 152 586 women were included in the study. A four-digit diagnostic code according to the seventh revision of the International Classification of Diseases (ICD-7) was used. The following ICD-7 codes were pooled: ‘upper aerodigestive tract’, codes 140–141, 143–148, and 161; ‘colorectum’, codes 153–154; ‘liver’, codes 155–156; ‘non-Hodgkin's lymphoma’, codes 200 and 202; and ‘leukaemia’, codes 204–207.

Our database for analysis of risk for second primary malignancies according to family history of breast cancer was created by a link between the Cancer Registry from 1958 to 2000 and Multigeneration Register using the individually unique national registration number. Statistics Sweden maintains a Multigeneration Register where offspring born in Sweden in 1932 and later are registered with their biological parents as families (Hemminki *et al*, 2001). At present, it includes 11 million individuals, who are structured in 3.1 millions of nuclear families. Breast cancer history was collected on all first-degree relatives, namely parents, siblings, and offspring, and if positive, this is described in this paper as ‘with a family history’.

### Statistical methods

Standardised incidence ratios (SIRs) were calculated by dividing the observed number of second primary cancers by the number expected based on population rates. The observed number of cases was assumed to be Poisson distributed, and 95% confidence intervals (CIs) for SIR were calculated (Breslow and Day, 1987). Person years at risk were calculated as starting at date of breast cancer diagnosis and ending at date of death, emigration, second primary cancer diagnosis, or 31 December 2000, whichever date came first. The expected number of cancers was estimated by multiplying the age-, sex-, and calendar year-specific rates from the Swedish Cancer Registry by the accumulated person years at risk. The follow-up period was divided into 1–9 years and 10 or more years after breast cancer diagnosis. The all sites SIRs combine the specific cancer sites presented in each table.

The SIRs for second primary malignancies according to family history of breast cancer were calculated similarly, except that the expected numbers of cancers were obtained using age-, sex-, and calendar year-specific rates in the corresponding general population in our database. The ratio of SIR with a family history to SIR without a family history was calculated to easily compare these two groups, and 95% CIs were estimated (Clayton and Hill, 1993). Similarly, the ratio of SIR for women <50 years of age at breast cancer diagnosis to that ⩾50 years was calculated.

## RESULTS

Table 1 presents SIRs by specific sites of subsequent cancers. A 22% increased risk of a second primary malignancy other than breast cancer was found. The largest significant excess risk was found for connective tissue cancer (SIR=1.78; 95% CI: 1.49–2.10). In addition, we investigated cancer sites stratified by time since breast cancer diagnosis, 1–9 and ⩾10 years (data not shown). Sites with SIRs that were significantly higher for the longer follow-up period were lung cancer (SIR=1.00, 95% CI=0.89–1.11, *n*=316 *vs* SIR=1.92, 95% CI=1.74–2.12, *n*=401) and non-Hodgkin's lymphoma (NHL) (SIR=0.93, 95% CI=0.80–1.06, *n*=188 *vs* SIR=1.42, 95% CI=1.23–1.64, *n*=202). In contrast, significantly elevated SIRs that were higher for the shorter follow-up period were observed for cancers of the endometrium (SIR=1.65, 95% CI=1.53–1.78, *n*=671 *vs* SIR=1.35, 95% CI=1.21–1.50, *n*=335) and central nervous system (SIR=1.25, 95% CI=1.10–1.41, *n*=254 *vs* SIR=1.00, 95% CI=0.82–1.20, *n*=113).

The effect of calendar period (1958–1979 *vs* 1980–2000) of breast cancer diagnosis was also studied (data not shown). In order to restrict the problem with truncation, the risk was estimated only for follow-up periods 1–9 and 10–14 years after breast cancer diagnosis. There were no apparent differences in risk by calendar period, even after taking time since breast cancer diagnosis into consideration, except for endometrial cancer where the risk in those followed for 10–14 years was higher for the later (SIR=1.67, 95% CI: 1.32–2.06, *n*=77) compared to the earlier (SIR=1.25, 95% CI: 0.99–1.54, *n*=79) period.

Standardised incidence ratios stratified by age (<50 and >50 years) for selected sites with a significantly elevated overall SIR and at least 100 observed cancers are presented in Table 2. Standardised incidence ratios were also calculated separately for subcategories of leukaemia and for connective tissue cancers in the thorax or upper limbs, areas more likely to be in the radiation treatment field. For most cancers, the risk decreased with increasing age at breast cancer diagnosis, the higher risk ratio for younger compared to older women for cancers at all sites combined being 1.36. Cancer sites where the significant ratio was higher than 1.50 were oesophagus (ratio 1.84), stomach (ratio 1.65), pancreas (ratio 1.51), lung (ratio 2.07), ovary (ratio 1.82), thyroid (ratio 1.62), and connective tissue (ratio 2.21), particularly thorax and upper limbs (ratio 2.32). A significantly higher risk of leukaemia (reflecting the patterns observed for acute and chronic myeloid leukaemia) was seen in younger compared to older women 1–9 years after diagnosis of breast cancer (SIR=2.86, 95% CI=1.90–4.02, *n*=28 *vs* SIR=1.28, 95% CI=1.10–1.47, *n*=185; ratio=2.23), but this difference diminished in the later follow-up period (SIR=1.38, 95% CI=0.95–1.94, *n*=33 *vs* SIR=1.33, 95% CI=1.08–1.63, *n*=97; ratio=1.04). A similar pattern was seen for connective tissue cancer (SIR=4.72, 95% CI=2.92–6.95, *n*=21 *vs* SIR=1.19, 95% CI=0.88–1.56, *n*=48; ratio=3.97), particularly of the thorax and upper limbs (SIR=11.11, 95% CI=5.51–18.65, *n*=11 *vs* SIR=2.79, 95% CI=1.77–4.05, *n*=23; ratio=3.98). For endometrial cancer, a statistically higher risk for older compared to younger women (SIR=1.15, 95% CI=0.86–1.47, *n*=55 *vs* SIR=1.72, 95% CI=1.59–1.86, *n*=616; ratio 0.67) was observed in the 1- to 9-year follow-up period.

Risks of second primary cancers for women with and without a family history of breast cancer are presented in Table 3. Significantly higher risks analysed as a ratio between women with and without a family history were seen for cancer of the oesophagus, stomach, ovary, NHL, and leukaemia. The highest SIRs were seen for second primary cancers of the oesophagus (SIR=7.54, 95% CI=2.38–15.60, *n*=5) and NHL (SIR=6.10, 95% CI=3.67–9.16, *n*=19) among women with a family history in the ⩾10 year follow-up period. Their respective ratios comparing women with and without a family history were statistically significant (5.89 and 4.33).

## DISCUSSION

Multiple malignancies may reflect increased surveillance, previous therapy, shared aetiological (e.g. lifestyle) factors, and genetic predisposition. By examining the influence of age at first cancer, family history of breast cancer, time since breast cancer, and their interaction, we aimed to explore the aetiology of second primary malignancies.

In our study, the SIRs for radiation-related solid tumours (e.g. oesophagus, lung, NHL, and connective tissue, particularly thorax and upper limbs) were higher in the 10 or more years follow-up period, whereas leukaemia risk was similar for both time periods. Women diagnosed before the age of 50 years had a substantially higher risk for all cancer sites (except the endometrium, central nervous system, acute and chronic lymphatic leukaemia, and melanoma), possibly due to increased susceptibility as well as more aggressive treatment for breast cancer in younger women. Increased risks analysed as the ratio between women with and without a family history of breast cancer were seen in cancers of the oesophagus, stomach, ovary, NHL, and leukaemia.

The almost six-fold higher risk of oesophagus cancer we found in women with a family history of breast cancer compared to women without a family history of breast cancer ⩾10 years following breast cancer diagnosis (SIR=7.54 *vs* 1.28) suggests an interaction between radiotherapy and inherited factors. In earlier studies, a high familial risk was found (Czene *et al*, 2002; EBCTCG, 2005). In addition, environmental factors such as tobacco and alcohol are important for oesophageal cancer and probably explain part of the familial clustering, although this could be due to chance, because the study was based on only five cases.

We found an increased risk of stomach cancer, which was significantly higher in women diagnosed with breast cancer before 50 and in women with a family history of breast cancer, suggesting a role of shared environmental and genetic factors. Rare syndromes such as Li–Fraumeni syndrome and ataxia telangiectasia can explain little of the elevated risk (Bevan and Houlston, 1999).

As in other studies (Neugut *et al*, 1994) and consistent with the effects of radiotherapy, risks of lung cancer were greater with longer follow-up time and younger age at breast cancer diagnosis. We recently reported an interaction between ionising radiation and smoking in women with breast cancer where the risk of lung cancer was confined to smokers who received radiotherapy (Prochazka *et al*, 2005).

We found the highest risk of endometrial cancer among women aged 50 or older at breast cancer diagnosis in the 1–9 year follow-up period. Our age and temporal findings (risk was higher during the later study period, 1980–2000) are consistent with the effects of tamoxifen treatment starting in the late 1970s in women over age 50 (Bergman *et al*, 2000; Swerdlow and Jones, 2005). An increased risk of breast cancer after endometrial cancer has also been reported (Mellemkjaer *et al*, 2006). Shared risk factors such as early menarche, late menopause, and nulliparity (Bergman *et al*, 2000) probably also contributed to our findings.

*BRCA-1* and *BRCA-2* genes are known to be involved in inherited susceptibility to breast and ovarian cancer (Lindor and Greene, 1998; Welcsh and King, 2001) and may contribute to the 1.8-fold higher risk of second ovarian cancer we observed in women diagnosed with breast cancer at younger ages as well as the 1.7-fold higher risk in women with a family history of breast cancer compared with those without a family history of breast cancer.

We found an increased risk of melanoma for women with a family history of breast cancer, suggesting genetic influence. A number of studies have suggested mutations of *CDKN2A* and *BRCA2* as possible determinants of the higher incidence of melanoma among breast cancer patients (Borg *et al*, 2000; Goggins *et al*, 2004).

We found a higher overall risk of a connective tissue malignancy with a longer follow-up time. However, when age and time since breast cancer diagnosis were considered jointly, an almost five-fold risk was observed in women <50 years old within the first 10 years following breast cancer diagnosis. The risk was even more pronounced in connective tissue cancers of the thorax or upper limbs, parts often included in the radiation field during breast cancer treatment, as previously reported (Harvey and Brinton, 1985; Kirova *et al*, 2005).

In agreement with previous reports, we found that the risk of NHL increased with time (Harvey and Brinton, 1985). In our study, the risk ⩾10 years after breast cancer diagnosis was four times higher in women with a family history of breast cancer compared to those without a family history, suggesting possible interaction of treatment with genetic factors.

A review (van Leeuwen and Travis, 2001) as well as later work (Smith *et al*, 2003; Raymond and Hogue, 2006) reported that alkylating agents/radiotherapy increased the risk of leukaemia beginning 1–2 years following treatment and peaking at 5–10 years after treatment, consistent with our results. We also found an elevated risk of leukaemia in women with a family history of breast malignancy.

The strengths of our study are the population-based design, complete follow-up of all patients, and the ability to identify women with a family history of breast cancer. We excluded malignancies diagnosed during the first year of follow-up avoiding detection of indolent tumours by increased surveillance. Among limitations we could not identify potential treatment effects because therapy information is not recorded in the cancer register.

In conclusion, we found that women diagnosed with breast cancer had a 22% increased risk of developing a second primary non-breast malignancy. Women with breast cancer before age 50 and women with a family history of breast cancer had elevated risks of developing several cancers, indicating a genetic predisposition to develop multiple tumours and/or susceptibility to the carcinogenic effect of breast cancer therapy.

## Figures and Tables

**Table 1:** Number of observed second primary cancers (Obs) following breast cancer, diagnosed between 1958 and 2000, standardised incidence ratios (SIRs), and 95% confidence interval (CI) among women

**Second primary cancer site**	**Obs**	**SIR**	**95% CI**
Upper aerodigestive tract	137	0.89	(0.75–1.04)
Salivary glands	43	**1.67**	**(1.21–2.20)**
Oesophagus	116	**1.57**	**(1.24–1.78)**
Stomach	686	**1.40**	**(1.30–1.51)**
Small intestine	76	**1.29**	**(1.02–1.60)**
Colorectum	1923	**1.15**	**(1.10–1.20)**
Liver	451	0.92	(0.84–1.01)
Pancreas	471	**1.12**	**(1.02–1.22)**
Lung	717	**1.37**	**(1.27–1.47)**
Cervix	217	0.92	(0.80–1.05)
Endometrium	1006	**1.54**	**(1.44–1.63)**
Ovary	712	**1.28**	**(1.19–1.38)**
Kidney	443	**1.31**	**(1.19–1.44)**
Urinary organs	383	1.10	(0.99–1.21)
Melanoma	360	**1.27**	**(1.14–1.40)**
Nervous system	367	**1.16**	**(1.05–1.28)**
Thyroid	164	**1.55**	**(1.32–1.80)**
Endocrine glands	270	**1.08**	**(1.96–1.22)**
Bone	21	**1.68**	**(1.04–2.48)**
Connective tissue	129	**1.78**	**(1.49–2.10)**
Non-Hodgkin's lymphoma	390	**1.13**	**(1.02–1.25)**
Hodgkin's disease	38	1.05	(0.74–1.41)
Multiple myeloma, plasmocytoma	182	1.04	(0.89–1.20)
Leukaemia	343	**1.37**	**(1.23–1.52)**
All the above sites	9758	**1.22**	**(1.19–1.24)**

Bold denotes statistical significance.

**Table 2:** Number of observed second primary cancers (Obs) following breast cancer, diagnosed between 1958 and 2000, standardised incidence ratios (SIRs), and 95% confidence interval (CI) for second primary malignancy according to age at breast cancer diagnosis

	**Age at breast cancer diagnosis**						
	**<50 years**			**⩾50 years**			
**Second primary cancer site**	**Obs**	**SIR**	**95% CI**	**Obs**	**SIR**	**95% CI**	**Ratio**
Oesophagus	20	**2.54**	(1.55–3.78)	96	**1.38**	(1.12–1.67)	1.84*
Stomach	97	**2.18**	(1.77–2.63)	589	**1.32**	(1.22–1.43)	1.65*
Colorectum	278	**1.38**	(1.23–1.58)	1645	**1.12**	(1.06–1.17)	1.24*
Pancreas	74	**1.60**	(1.26–1.99)	397	1.06	(0.96–1.16)	1.51*
Lung	235	**2.35**	(2.06–2.66)	482	**1.14**	(1.04–1.24)	2.07*
Endometrium	183	**1.29**	(1.11–1.49)	823	**1.61**	(1.50–1.72)	0.80*
Ovary	247	**1.97**	(1.73–2.22)	465	1.08	(0.98–1.18)	1.82*
Kidney	74	**1.50**	(1.18–1.86)	369	**1.28**	(1.15–1.41)	1.17
Melanoma	91	**1.32**	(1.06–1.61)	269	**1.25**	(1.10–1.40)	1.06
Nervous system	89	1.25	(1.00–1.52)	278	**1.14**	(1.01–1.27)	1.10
Thyroid	51	**2.21**	(1.65–2.86)	113	**1.36**	(1.12–1.63)	1.62*
Endocrine glands	75	**1.36**	(1.07–1.68)	195	1.01	(0.87–1.15)	1.35*
Connective tissue	40	**3.27**	(2.33–4.36)	89	**1.48**	(1.19–1.80)	2.21*
Thorax and upper limbs	19	**8.02**	(4.82–12.03)	42	**3.46**	(2.49–4.58)	2.32*
Non-Hodgkin's lymphoma	77	**1.51**	(1.19–1.87)	313	1.06	(0.95–1.19)	1.42*
Leukaemia	61	**1.81**	(1.38–2.29)	282	**1.30**	(1.15–1.46)	1.39*
Acute lymphatic leukaemia	2	1.20	(0.14–3.45)	20	**2.19**	(1.34–3.26)	0.55
Chronic lymphatic leukaemia	14	1.15	(0.63–1.84)	87	0.96	(0.77–1.17)	1.21
Acute myeloid leukaemia	22	**1.97**	(1.23–2.88)	91	**1.46**	(1.17–1.77)	1.35
Chronic myeloid leukaemia	13	**2.89**	(1.53–4.67)	33	**1.56**	(1.08–2.14)	1.85
All the above sites	1692	**1.64**	(1.56–1.72)	6405	**1.20**	(1.17–1.23)	1.36*

Standardised incidence ratio for women <50 years of age at breast cancer diagnosis was compared with SIR for women ⩾50 years of age at breast cancer diagnosis (ratio).

Bold denotes statistical significance

* denotes statistically significant ratio.

**Table 3:** Number of observed second primary cancers (Obs) following breast cancer, diagnosed between 1958 and 2000, standardised incidence ratios (SIRs), and 95% confidence interval (CI) for second primary malignancy according to family history of breast cancer

	**Family history of breast cancer**						
	**Yes**			**No**			
**Second primary cancer site**	**Obs**	**SIR**	**95% CI**	**Obs**	**SIR**	**95% CI**	**Ratio**
Oesophagus	7	**3.76**	**(1.49–7.07)**	58	**1.69**	**(1.29–2.16)**	2.22*
Stomach	27	**2.65**	**(1.74–3.74)**	296	**1.41**	**(1.26–1.58)**	1.88*
Colorectum	46	**1.10**	**(0.81–1.45)**	1010	**1.18**	**(1.11–1.25)**	0.93
Pancreas	9	0.86	(0.39–1.52)	253	**1.18**	**(1.04–1.33)**	0.73
Lung	16	0.91	(0.52–1.41)	483	**1.45**	**(1.32–1.58)**	0.63
Endometrium	41	**1.94**	**(1.39–2.58)**	589	**1.45**	**(1.34–1.57)**	1.33
Ovary	40	**2.32**	**(1.66–3.10)**	437	**1.38**	**(1.25–1.51)**	1.68*
Kidney	8	0.89	(0.38–1.61)	239	**1.29**	**(1.14–1.46)**	0.69
Melanoma	18	**1.67**	**(1.00–2.53)**	231	**1.28**	**(1.12–1.45)**	1.31
Nervous system	13	1.20	(0.63–1.94)	236	**1.25**	**(1.09–1.41)**	0.96
Thyroid	5	1.51	(0.48–3.12)	104	**1.78**	**(1.46–2.14)**	0.85
Endocrine glands	12	1.37	(0.70–2.25)	199	**1.19**	**(1.03–1.36)**	1.15
Connective tissue	5	2.48	(0.78–5.12)	79	**2.06**	**(1.63–2.54)**	1.20
Non-Hodgkin's lymphoma	23	**2.41**	**(1.52–3.49)**	231	1.13	(0.99–1.28)	2.13*
Leukaemia	19	**2.25**	**(1.35–3.38)**	218	**1.29**	**(1.13–1.47)**	1.74*
All the above sites	289	**1.58**	**(1.40–1.77)**	4663	**1.31**	**(1.27–1.35)**	1.21*

Standardised incidence ratio for women with family history of breast cancer was compared with SIR for women without family history of breast cancer (ratio).

Bold denotes statistical significance; ^*^ denotes statistically significant ratio.
